# Factors associated with health-related quality of life in Korean older workers

**DOI:** 10.1186/s40557-015-0077-9

**Published:** 2015-11-30

**Authors:** Sujin Hong, Harin Jeong, Yunjeong Heo, Hosun Chun, Jongtae Park, Daeseong Kim

**Affiliations:** Department of Occupational Medicine, Korea University Hosipital, 123, Jeokgeum-ro, Ansan, Gyeonggido Republic of Korea

**Keywords:** Older workers, Health-related quality of life, EQ-5D, KNHANES

## Abstract

**Objectives:**

The prevalence of aged individuals in the Korean workforce continues to increase. This research determined the health and working conditions of Korean older wage workers and confirmed the effects of factors on the health-related quality of life of Korean older workers.

**Methods:**

Of the 25,534 persons surveyed in the fifth Korea National Health and Nutrition Examination Survey, 1368 older (>55 years of age) wage workers without missing variables were selected. Their general characteristics, health status (cardiovascular disease, musculoskeletal disease, and mental health), working conditions (type of occupation, employment status, full- or part-time work, weekly average working hours, and shift work), and health-related quality of life assessed by the EQ-5D questionnaire were examined.

**Results:**

The mean values of the EQ-5D index of the male and female older workers were 0.956 ± 0.087 and 0.917 ± 0.124, respectively (*p* < 0.001). The factors that caused statistically significant differences in the EQ-5D index for all subjects were age, education, household income, cerebro-cardiovascular event, osteoarthritis, musculoskeletal pain, stress, occupation type, employment status, and working hours. In logistic regression analysis, the factors that associated with perceived problems in each EQ-5D dimensions were age, musculoskeletal pain, stress, diabetes, smoking, occupation type, employment status, and working hours.

**Conclusions:**

To eventually raise the quality of life of older workers through health maintenance and management, it is necessary to manage related factors that include of musculoskeletal pain and diseases, stress, diabetes, smoking, occupation, employment status, and working hours.

## Background

An aging society is defined as a prevalence of >7 % of those ≥65 years of age [1]. The prevalence of this age group in an aged society and super-aged society is >14 and >20 %, respectively [1]. By these definitions, Korea is an aging society, with 7.25 % of the total population ≥65 years of age in 2000 [2]. By 2017–2018, Korea will become a post-aged society, with the prevalence of aged Koreans rising to 14.0 % (Statistics Korea, 2011 [3]).

Of note, the economically active segment of Korean society <65 years of age is shrinking. As Korean society continues to disproportionately age, workers in the 30s who represent the most economically active segment of society will further diminish. By 2025, the most economically active segment of Korean society will be those 50–60 years of age [3].

The overall decrease in manpower and the decline in productivity due to societal aging portend serious social problems in Korea that include elderly poverty, which can diminish welfare and quality of life of the elderly. To prevent a rapid decrease in the workforce, countermeasures to raise the rate of participation in overall economic activities and rate of employment should be formulated. As well, measures need to be taken to maintain an elderly workforce and to vitalize employment.

Studies of older workers have focused mainly on industrial accidents and injuries [4–7]. But the prevalence of chronic diseases also increases, which is another factor in the exit from the workforce. To maintain the working ability of workers, their health should be managed. To achieve this, older workers’ health status first needs to be determined.

Also, as the period of old age grows longer, the quality of life of the aged must be enhanced beyond the simple goal of survival. In particular, the health-related quality of life (HRQL) of the aged must be improved.

Measuring the HRQL of a population group is important because such data can be used as evidence in making decisions on health and medical treatment policies, and can be indicators of the outcomes of related projects. Many studies have been conducted in the past 10 years in Korea on the relationships between HRQL and a number of factors including disease [8–12], sex [13], age [11, 13–16], income bracket [16, 17], daily life skills [15], subjective health awareness and health behavior [18]. However, few studies have involved older workers from the viewpoint of occupation.

Using the fifth (2010 ~ 2012) Korea National Health and Nutrition Examination Survey, (KNHANES) we report the current health status and working conditions of older workers of Korea, and confirm the effects of the aforementioned factors on the HRQL of older workers.

## Materials and methods

### Subjects

Of the 25,534 participants, 6383 wage workers were selected. From these wage workers, older workers ≥55 years of age (*n* = 1413) were selected based on the criterion presented in the Employment Promotion for the Aged Act and the Employment Protection Act. Forty five individuals were excluded for missing sociodemographic factor values (*n* = 15), missing health-related factors (*n* = 14), missing working condition-related values (*n* = 12), and missing health status and HRQL values (*n* = 4). The remaining 1368 persons were analyzed.

### Methods

#### General characteristics

Sociodemographic factors included sex, age, education, household income, and marital status (having a spouse). Age groups were divided into 55–64 years, 65–74 years, and ≥75 years. Level of education was classified into elementary school graduate and below, middle school graduate, high school graduate, and college graduate and above. Income level according to household income quartile was classified into low, below middle, above middle, and high. Drinking (none, 1–4 times a month, >2 times a week), smoking (current smoker, ex-smoker, non-smoker), and obesity (body mass index 18.5, 18.5–22.9, 23.0–24.9, 25.0–29.9, ≥30.0 kg/m^2^) were classified respectively.

#### Health status

Chronic diseases were examined because of their appreciable effects on older workers’ health status. Questions concerned cardiovascular diseases, musculoskeletal diseases, and mental health. Cardiovascular diseases included the presence of hypertension, hyperlipidemia, stroke, myocardial infarction, angina, and diabetes. The prevalence of diseases like diabetes was determined based on the reported diagnosis. Musculoskeletal diseases or symptoms included the presence of osteoarthritis and knee, hip and back pain, with subjects queried whether they had experienced knee, hip and back pain for 30 days or more in the prior 3 months. Assessment of mental health status included the presence of depression and stress recognition rate. The stress recognition rate was classified based on responses to the occurrence of daily stress (high = very much or much, low = little or rarely).

#### Working conditions

Types of occupation, types of employment by employment status and working time, weekly average working hours, and shift work were examined. The types of occupation were classified into office work (administrators, specialists and related workers, and office workers), service work (service and sales employees), manufacturing work (skilled agriculture workers, forestry and fishery workers, functional employees and related functional employees, and equipment, machine operation, and assembly workers), and elementary occupations. The subjects were classified according to their employment status into regular employees, temporary employees, and daily employees, and whether they worked part-time or full-time. The weekly average working hours was classified as <40, 40–52, 52–60, and <60. Shift work, except for day duty workers, comprised all workers who worked in the afternoon (2 p.m. to midnight), at night (9 p.m. to 8 a.m.), in regular rotation of shifts between the day and night shifts, in 24-h shifts, in segmented shifts (working more than two shifts a day), and in irregular shifts.

#### HRQL

The World Health Organization (WHO) defines quality of life as the degree of realization of a person’s interests, expectations, ideals, and hopes according to his or her current value and cultural system, in his or her life [19, 20]. Quality of life is so comprehensive and extensive that it is sometimes classified into non-health-related quality of life (NHRQL) and HRQL. HRQL represents general well-being as well as elements that have direct effects on the individual’s physical, psychological, and mental health [21]. There are many instruments for evaluating HRQL. They include the QWB scale [22], HUI-II [23] and –III [24], EQ-5D [25], and SF-6D [26]. EQ-5D, developed by the EuroQoL Group to measure general health status, has advantages that include simple questions, applicability to diverse clinical situations, and easy and quick preparation [25]. The fifth KNHANES also used EQ-5D to measure HRQL. The analysis in this study was conducted based on the results of the survey. In the survey, the subjects were asked to choose one of the following three responses, for each of the five given dimensions, that best explained their current health status: “1 = no problem,” “2 = some problems”, and “3 = severe problems”. The five questions concerning health status expressed health status between 1, which represents perfect health status, and −1, which represents a health status that is no better than death. In this research, the EQ-5D index, which Nam et al. calculated using their estimated weighted quality value for Koreans [27], was used. The formula for the EQ-5D index is:

EQ-5D index = 1 - (0.05 + 0.096 × M2 + 0.418 × M3 + 0.046 × SC2 + 0.136 × SC3 + 0.051 × UA2 + 0.208 × UA3 + 0.037 × PD2 + 0.151 × PD3 + 0.043 × AD2 + 0.158 × AD3 + 0.05 × N3).

where M2 - Mobility “level 2” = 1; otherwise, 0; M3 - Mobility “level 3” = 1; otherwise, 0; SC2 - Self-care “level 2” = 1; otherwise, 0; SC3 - Self-care “level 3” = 1; otherwise, 0; UA2 - Usual activities “level 2” = 1; otherwise, 0 ; UA3 - Usual activities “level 3” = 1; otherwise, 0; PD2 - Pain/discomfort “level 2” = 1; otherwise, 0; PD3 - Pain/discomfort “level 3” = 1; otherwise, 0; AD2 - Anxiety/depression “level 2” = 1; otherwise, 0; AD3 - Anxiety/depression “level 3” = 1; otherwise, 0; N3 - Only one “level 3” = 1, and the rest = 0.

### Data analysis

The subjects’ characteristics were presented using their frequency and percentage. To compare the HRQL values according to the subjects’ sociodemographic characteristics, working conditions, and health status, t-test and ANOVA were used with a statistical significance level of α = 0.05. To adjust for the effect of confounders and to understand reciprocal action, logistic regressions were conducted. Because there is no standard point that can be used as a standard dichotomy in EQ-5D index, and EQ-5D index score is calculated by using their estimated weighted quality value of response to each of the five dimensions, and the weighted quality values are different for each country, we concluded that it is a more fundamental and concrete method to see response to each dimension rather than EQ-5D index score in logistic regression analysis. We used the response of EQ-5D dimensions as dependent variable. For all 5 dimensions level 2 and 3 on the EQ-5D dimensions were merged and thus dichotomized to “no problem”(0: level 1) or “some or extreme problem” (1: level 2 and 3). All analyses were performed in SPSS version 18.0 (SPSS, Chicago, IL, USA).

## Results

### General characteristics, health status, and working conditions distribution

Of the 1368 older wage workers, 712 (52 %) were male and 656 (48 %) were female. Workers 55–64 years of age comprised the majority of older age workers (64.7 % males, 60.4 % female). The percentages decreased with increasing age, with the respective percentage of males and females being 30.9 and 32.2 % in the 65–74 age group, and 4.4 and 7.5 % in the ≥74 year old group. Of all male and female older wage workers, the highest percentage groups in education level were high school graduates (32.4 %) and elementary school graduates (63.7 %), respectively. Concerning household income quartile, of the male and female older workers, the highest percentage was in the 2nd quartile (32.3 %) and 1st quartile (36.3 %), respectively (Table 1).

**Table 1:** General characteristics and health status of older wage workers, according to sex

	Total (%)	M (%)	F (%)
	1368 (100.0)	712 (52.0)	656 (48.0)
Age			
55–64	857 (62.6)	461 (64.7)	396 (60.4)
65–74	431 (31.5)	220 (30.9)	211 (32.2)
75-	80 (5.8)	31 (4.4)	49 (7.5)
Education			
< Elementary school	609 (44.5)	191 (26.8)	418 (63.7)
Middle school	263 (19.2)	139 (19.5)	124 (18.9)
High school	316 (23.1)	231 (32.4)	85 (13.0)
> College	180 (13.2)	151 (21.2)	29 (4.4)
Household Income (quartile)			
1st (lowest)	344 (25.1)	106 (14.9)	238 (36.3)
2nd	424 (31.0)	230 (32.3)	194 (29.6)
3rd	309 (22.6)	186 (26.1)	123 (18.8)
4th (highest)	291 (21.3)	190 (26.7)	101 (15.4)
Existence of spouse			
Yes	1123 (82.1)	676 (94.9)	447 (68.1)
No	245 (17.9)	36 (5.1)	209 (31.9)
Drinking			
none	448 (32.7)	137 (19.2)	311 (47.4)
1 ~ 4/month	599 (43.8)	295 (41.4)	304 (46.3)
2~/week	321 (23.5)	280 (39.3)	41 (6.3)
Smoking			
non-smoker	749 (54.8)	128 (18.0)	621 (94.7)
ex-smoker	395 (28.9)	378 (53.1)	17 (2.6)
current smoker	224 (16.4)	206 (28.9)	18 (2.7)
BMI			
< 18.5	35 (2.6)	22 (3.1)	13 (2.0)
18.5–22.9	493 (36.0)	269 (37.8)	224 (34.1)
23.0 ~ 24.9	391 (28.6)	197 (27.7)	194 (29.6)
25.0–29.9	413 (30.2)	213 (29.9)	200 (30.5)
30.0-	36 (2.6)	11 (1.5)	25 (3.8)
Hypertension			
Yes	523 (38.2)	266 (37.4)	257 (39.2)
No	845 (61.8)	446 (62.6)	399 (60.8)
Hyperlipidemia			
Yes	228 (16.7)	105 (14.7)	123 (18.8)
No	1140 (83.3)	607 (85.3)	533 (81.3)
Cerebro-cardiovascular event (Stroke, Myocardial infarction, Angina)			
Yes	90 (6.6)	49 (6.9)	41 (6.3)
No	1278 (93.4)	663 (93.1)	615 (93.8)
Diabetes			
Yes	170 (12.4)	108 (15.2)	62 (9.5)
No	1198 (87.6)	604 (84.8)	594 (90.5)
Osteoarthritis			
Yes	249 (18.2)	59 (8.3)	190 (29.0)
No	1119 (81.8)	653 (91.7)	466 (71.0)
Musculoskeletal pain (Knee, Hip, Back)			
Yes	434 (31.7)	137 (19.2)	297 (45.3)
No	934 (68.3)	575 (80.8)	359 (54.7)
Depression			
Yes	54 (3.9)	13 (1.8)	41 (6.3)
No	1314 (96.1)	699 (98.2)	615 (93.8)
Stress			
High	264 (19.3)	115 (16.2)	149 (22.7)
Low	1104 (80.7)	597 (83.8)	507 (77.3)

Concerning smoking behavior, the percentage of ex-smokers among the male older workers was the highest (53.1 %). Among females, the percentage of non-smokers was the highest (94.7 %). The most frequent health problem of male older workers was hypertension (37.4 %), followed by musculoskeletal pain (19.2 %), stress (16.2 %), diabetes (15.2 %), and hyperlipidemia (14.7 %). The most frequent health problem of female older workers was musculoskeletal pain (45.3 %), followed by hypertension (39.7 %), osteoarthritis (29.0 %), stress (22.7 %), and hyperlipidemia (18.8 %) (Table 1).

Regarding the distribution of occupations, among the male older workers, the percentage of elementary occupations was the highest at 42.0 %, followed by office work (28.7 %), manufacturing work (26.0 %), and service work (6.4 %). Among female older workers, the percentage of elementary occupations was also the highest at 66.0 %, followed by service work (20.4 %), office work (9.5 %), and manufacturing work (4.1 %). Concerning the employment status, over half of the male older workers were regular employees, with more females having temporary and daily employment than regular employment. The percentage of part-time workers was higher (36.4 %) among the female older workers than males (11.9 %). The average number of weekly working hours of male and female older workers was 45.00 ± 21.18 and 31.63 ± 19.37, respectively. The percentage of male older workers with 40–52 weekly working hours was the highest at 36.2 %, and the percentage of female older workers with less than 40 weekly working hours was the highest at 60.4 %. The percentage of shiftwork was higher among the male older workers (29.5 %) than among female older workers (13.1 %) (Table 2).

**Table 2:** Working conditions of older wage workers according to sex

	Total (%)	M (%)	F (%)
Occupation			
Office work	266 (19.4)	204 (28.7)	62 (9.5)
Service work	158 (11.5)	24 (3.4)	134 (20.4)
Manufacturing work	212 (15.5)	185 (26.0)	27 (4.1)
Elementary occupations	732 (53.5)	299 (42.0)	433 (66.0)
Employment status			
Regular employee	756 (55.3)	478 (67.1)	278 (42.4)
Temporary employee	305 (22.3)	121 (17.0)	184 (28.0)
Daily employee	307 (22.4)	113 (15.9)	194 (29.6)
Full-/Part -Time			
Full time worker	1044 (76.3)	627 (88.1)	417 (63.6)
Part time worker	324 (23.7)	85 (11.9)	239 (36.4)
Working hour /week			
Mean ± S.D°	38.60 ± 21.40	45.00 ± 21.18	31.63 ± 19.37
< 40	623 (45.5)	227 (31.9)	396 (60.4)
40 ~ 52	426 (31.1)	258 (36.2)	168 (25.6)
52 ~ 60	87 (6.4)	54 (7.6)	33 (5.0)
≥60	232 (17.0)	173 (24.3)	59 (9.0)
Shift work			
Yes	1072 (78.4)	210 (29.5)	86 (13.1)
No	296 (21.6)	502 (70.5)	570 (86.9)
Total	1368 (100.0)	712 (100.0)	656 (100.0)

*SD* standard deviation

### HRQL

EQ-5D index scores for male and female older workers were 0.956 ± 0.087 and 0.917 ± 0.124, respectively; the difference was significant (*p* < 0.001). As age increased for both genders the EQ-5D index significantly decreased (*p* < 0.001). There were significant differences in the EQ-5D index according to the level of education. Among the male older workers, increasing level of education was associated with increased EQ-5D index (*p* < 0.001). Among the female older workers, the scores of elementary school graduates were the lowest; scores did not tend to increase according to the education level. For both genders, the EQ-5D index tended to significantly increase according to the income level (*p* < 0.001). Smoking behavior showed statistically significant differences in the EQ-5D index of the male older workers; EQ-5D index of the smokers was significantly lower than that of the non-smokers and ex-smokers (*p* < 0.001). The health status items associated with statistically significant differences in the EQ-5D index of both the male and female older workers were cerebro-cardiovascular events, osteoarthritis, musculoskeletal pain, and stress (Table 3).

**Table 3:** General characteristics, health status, and health-related quality of life instruments (EQ-5D index) of older wage workers, according to sex

	M			F		
	N	Mean ± S.D°	*P*-value	N	Mean ± S.D°	*P*-value
	712	0.956 ± 0.087		656	0.917 ± 0.124	0.000
Age						
55–64	461	0.966 ± 0.082	0.000	396	0.936 ± 0.106	0.000
65–74	220	0.940 ± 0.091		211	0.893 ± 0.139	
75-	31	0.925 ± 0.094		49	0.863 ± 0.152	
Education						
<Elementary school	191	0.939 ± 0.093	0.000	418	0.899 ± 0.137	0.000
Middle school	139	0.944 ± 0.119		124	0.953 ± 0.081	
High school	231	0.963 ± 0.069		85	0.937 ± 0.098	
> College	151	0.978 ± 0.055		29	0.958 ± 0.070	
Household Income (quartile)						
1st (lowest)	106	0.919 ± 0.138	0.000	238	0.882 ± 0.147	0.000
2nd	230	0.950 ± 0.083		194	0.928 ± 0.109	
3rd	186	0.968 ± 0.062		123	0.944 ± 0.101	
4th (highest)	190	0.972 ± 0.065		101	0.944 ± 0.095	
Existence of spouse						
Yes	676	0.956 ± 0.087	0.753	447	0.929 ± 0.112	0.001
No	36	0.952 ± 0.084		209	0.890 ± 0.142	
Drinking						
none	137	0.957 ± 0.088	0.866	311	0.906 ± 0.132	0.082
1 ~ 4/month	295	0.958 ± 0.093		304	0.928 ± 0.112	
2~/week	280	0.954 ± 0.079		41	0.924 ± 0.134	
Smoking						
non-smoker	128	0.969 ± 0.066	0.001	621	0.915 ± 0.125	0.416
ex-smoker	378	0.961 ± 0.073		17	0.953 ± 0.065	
current smoker	206	0.938 ± 0.114		18	0.932 ± 0.127	
BMI						
< 18.5	22	0.968 ± 0.072	0.701	13	0.912 ± 0.114	0.612
18.5–22.9	269	0.952 ± 0.084		224	0.922 ± 0.112	
23.0 ~ 24.9	197	0.958 ± 0.079		194	0.922 ± 0.129	
25.0–29.9	213	0.960 ± 0.097		200	0.905 ± 0.133	
30.0-	11	0.934 ± 0.107		25	0.924 ± 0.104	
Hypertension						
Yes	266	0.954 ± 0.079	0.684	257	0.903 ± 0.127	0.027
No	446	0.957 ± 0.091		399	0.926 ± 0.120	
Hyperlipidemia						
Yes	105	0.954 ± 0.073	0.819	123	0.901 ± 0.151	0.179
No	607	0.956 ± 0.089		533	0.921 ± 0.116	
Cerebro-cardiovascular event (Stroke, Myocardial infarction, Angina)						
Yes	49	0.915 ± 0.110	0.008	41	0.848 ± 0.186	0.017
No	663	0.959 ± 0.084		615	0.921 ± 0.117	
Diabetes						
Yes	108	0.940 ± 0.089	0.040	62	0.887 ± 0.161	0.122
No	604	0.959 ± 0.086		594	0.920 ± 0.119	
Osteoarthritis						
Yes	59	0.920 ± 0.092	0.001	190	0.860 ± 0.160	0.000
No	653	0.959 ± 0.085		466	0.940 ± 0.096	
Musculoskeletal pain (Knee, Hip, Back)						
Yes	137	0.892 ± 0.132	0.000	297	0.858 ± 0.149	0.000
No	575	0.971 ± 0.063		359	0.966 ± 0.065	
Depression						
Yes	13	0.941 ± 0.099	0.534	41	0.865 ± 0.145	0.005
No	699	0.956 ± 0.086		615	0.920 ± 0.121	
Stress						
High	115	0.942 ± 0.080	0.049	149	0.878 ± 0.153	0.000
Low	597	0.959 ± 0.088		507	0.928 ± 0.111	

*SD* standard deviation

The EQ-5D index according to the occupation was highest in the service work (0.984 ± 0.038) among the male older workers, and in the office work (0.984 ± 0.038) among the female older workers. Elementary occupations among both the male and female older workers had the lowest scores, which significantly differed from the highest scores. For the employment status, the EQ-5D index was significantly high among both the male and female regular employees (*p* < 0.001). For the working hours, the EQ-5D index was lowest in the group of males and females who worked for less than 40 h weekly (Table 4).

**Table 4:** Working conditions and health-related quality of life instruments (EQ-5D index) of older wage workers

	M			F		
	N	Mean ± S.D°	*P*-value	N	Mean ± S.D°	*P*-value
Occupation						
Office work	204	0.975 ± 0.058	0.000	62	0.961 ± 0.065	0.008
Service work	24	0.984 ± 0.038		134	0.927 ± 0.133	
Manufacturing work	185	0.962 ± 0.078		27	0.928 ± 0.096	
Elementary occupations	299	0.938 ± 0.105		433	0.907 ± 0.127	
Employment status						
Regular employee	478	0.966 ± 0.067	0.000	278	0.941 ± 0.088	0.000
Temporary employee	121	0.942 ± 0.097		184	0.900 ± 0.153	
Daily employee	113	0.928 ± 0.131		194	0.898 ± 0.130	
Full-/Part -Time						
Full time worker	627	0.957 ± 0.079	0.481	417	0.920 ± 0.119	0.009
Part time worker	85	0.950 ± 0.129		239	0.911 ± 0.131	
Working hour /week						
< 40	227	0.946 ± 0.106	0.043	396	0.905 ± 0.133	0.024
40 ~ 52	258	0.967 ± 0.069		168	0.930 ± 0.112	
52 ~ 60	54	0.965 ± 0.085		33	0.940 ± 0.090	
≥ 60	173	0.952 ± 0.081		59	0.945 ± 0.095	
Shift work						
Yes	210	0.957 ± 0.080	0.788	86	0.932 ± 0.096	0.217
No	502	0.956 ± 0.089		570	0.915 ± 0.127	

*SD* standard deviation

The percentage of male older workers who reported problems related to ‘mobility’, ‘self-care’, ‘usual activities’, ‘pain/discomfort’, and ‘anxiety/depression’ was 13.4, 3.5, 4.6, 17.3, and 7.7 %, respectively. The respective percentages for female older workers were 27.1, 4.4, 11.9, 30.5, and 15.4 % (Table 5).

**Table 5:** Distribution of levels of perceived problem in each of the dimensions of the EQ-5D descriptive system, according to sex in older wage workers

	M (*n* = 712)			F (*n* = 656)		
	Level of perceived problem (%)					
Dimension	1^a^	2^a^	3^a^	1^a^	2^a^	3^a^
Mobility	616 (86.5)	95 (13.3)	1 (0.1)	478 (72.9)	176 (26.8)	2 (0.3)
Self-care	694 (97.5)	15 (2.1)	3 (0.4)	627 (95.6)	27 (4.1)	2 (0.3)
Usual activities	679 (95.4)	32 (4.5)	1 (0.1)	578 (88.1)	74 (11.3)	4 (0.6)
Pain/discomfort	589 (82.7)	113 (15.9)	10 (1.4)	456 (69.5)	178 (27.1)	22 (3.4)
Anxiety/depression	657 (92.3)	54 (7.6)	1 (0.1)	555 (84.6)	93 (14.2)	8 (1.2)

^a^Level 1 implies no problem, 2 moderate problem, 3 severe problem

### Regression analyses

Logistic regression analysis was done on chosen variables (age, education, household income, smoking, hypertension, diabetes, musculoskeletal pain, stress, occupation, working hours, and employment status) which showed meaningful difference by EQ-5D index score. In the case of the male older workers (Table 6), age associated problems in all the dimensions (OR 1.03–1.07) and musculoskeletal pain had effects in all the dimensions except in the ‘anxiety/depression’ dimensions (OR 3.45–7.01). And education (OR 0.55, 95 % CI 0.33–0.93), smoking (OR 2.36, 95 % CI 1.45–3.85), diabetes (OR 1.94, 95 % CI 1.05–3.85), and occupation had statistically significant effects in the ‘mobility’ dimension (OR 0.38, 95 % CI 0.22–0.64). The employment status had effects in the ‘usual activities’ dimension (OR 2.78, 95 % CI 1.11–6.96); the type of occupation, in the ‘pain/discomfort’ dimension (OR 0.60, 95 % CI 0.38–0.95); and education and stress, in the ‘anxiety/depression’ dimension. In the case of the female older workers (Table 7), age and musculoskeletal pain had statistically significant effects in all dimensions (OR 1.02–1.07 and OR 2.62–12.43, respectively). The working hours had statistically significant effects in all dimensions except in the ‘anxiety/depression’ dimensions (OR 0.98–0.98). Stress had significant effects in the ‘self-care’, ‘usual activities’ and ‘anxiety/depression’ dimensions (OR 2.31, 2.34 and 4.09, respectively).

**Table 6:** Logistic regression analyses of the responses to the EQ-5D items by the male older workers

	Mobility	Self-care	Usual activities	Pain/discomfort	Anxiety/depression
	OR (95 % CI)	OR (95 % CI)	OR (95 % CI)	OR (95 % CI)	OR (95 % CI)
Age	1.03 (1.04 ~ 1.01)***	1.06 (1.1 ~ 1.03)***	1.07 (1.1 ~ 1.04)***	1.03 (1.05 ~ 1.02)***	1.05 (1.07 ~ 1.03)***
Education (/<Elementary school)	0.55 (0.33 ~ 0.93)*	0.57 (0.21 ~ 1.57)	0.66 (0.31 ~ 1.44)	0.80 (0.50 ~ 1.27)	0.52 (0.28 ~ 0.98)*
Household Income (quartile) (/4th(highest))					
1st (lowest)	0.96 (0.41 ~ 2.26)	1.97 (0.39 ~ 10.08)	2.21 (0.54 ~ 9.02)	1.84 (0.85 ~ 4.01)	2.02 (0.64 ~ 6.33)
2nd	0.78 (0.38 ~ 1.60)	0.86 (0.19 ~ 3.92)	1.96 (0.56 ~ 6.91)	1.62 (0.86 ~ 3.05)	2.42 (0.95 ~ 6.20)
3rd	0.71 (0.34 ~ 1.51)	0.19 (0.02 ~ 1.96)	0.87 (0.2 ~ 3.78)	1.17 (0.61 ~ 2.23)	1.56 (0.61 ~ 3.98)
Smoking (current/non & ex-smoker)	2.36 (1.45 ~ 3.85)***	1.11 (0.40 ~ 3.07)	1.96 (0.92 ~ 4.17)	1.14 (0.73 ~ 1.79)	0.79 (0.41 ~ 1.50)
Hypertension (/no)	1.13 (0.68 ~ 1.88)	0.74 (0.24 ~ 2.24)	1.28 (0.57 ~ 2.87)	1.30 (0.84 ~ 2.01)	1.26 (0.68 ~ 2.33)
Diabetes (/no)	1.94 (1.05 ~ 3.58)*	0.19 (0.02 ~ 1.56)	1.41 (0.53 ~ 3.76)	1.30 (0.74 ~ 2.27)	1.62 (0.78 ~ 3.36)
Musculoskeletal pain (/no)	7.01 (4.26 ~ 11.56)***	3.45 (1.28 ~ 9.34)*	5.82 (2.74 ~ 12.36)***	5.26 (3.38 ~ 8.17)***	1.62 (0.84 ~ 3.12)
Stress (/low)	0.79 (0.40 ~ 1.56)	2.12 (0.69 ~ 6.54)	0.32 (0.09 ~ 1.20)	1.18 (0.68 ~ 2.05)	4.95 (2.71 ~ 9.03)***
Occupation (/Elementary occupations)	0.38 (0.22 ~ 0.64)***	0.35 (0.11 ~ 1.13)	0.49 (0.22 ~ 1.10)	0.60 (0.38 ~ 0.95)*	0.73 (0.39 ~ 1.38)
Working hour/week	1.00 (0.98 ~ 1.01)	1.01 (0.99 ~ 1.04)	1.00 (0.98 ~ 1.02)	1.00 (0.99 ~ 1.01)	1.00 (0.98 ~ 1.01)
Employment status (/Regular employee)	1.20 (0.67 ~ 2.17)	1.97 (0.59 ~ 6.57)	2.78 (1.11 ~ 6.96)*	1.03 (0.62 ~ 1.73)	1.25 (0.62 ~ 2.52)
R^2^_N_	0.702	0.927	0.882	0.579	0.789

Some variables were reclassified because of small group size. Education (0: elementary school, 1: middle and high school, and collage); occupation (0: elementary occupations, 1: office, service and manufacturing work)

*OR* odds ratio, *CI* confidence intervals

*p < 0.05, ***p < 0.001

**Table 7:** Logistic regression analyses of the responses to the EQ-5D items by the female older workers

	Mobility	Self-care	Usual activities	Pain/discomfort	Anxiety/depression
	OR (95 % CI)	OR (95 % CI)	OR (95 % CI)	OR (95 % CI)	OR (95 % CI)
Age	1.02 (1.04 ~ 1.01)***	1.07 (1.1 ~ 1.04)***	1.06 (1.08 ~ 1.04)***	1.02 (1.03 ~ 1.01)***	1.04 (1.05 ~ 1.02)***
Education (/<Elementary school)	0.64 (0.40 ~ 1.02)	0.37 (0.12 ~ 1.15)	0.61 (0.31 ~ 1.21)	0.9 (0.59 ~ 1.40)	0.92 (0.54 ~ 1.58)
Household Income (quartile) (/4th(highest))					
1st (lowest)	1.43 (0.71 ~ 2.87)	1.42 (0.32 ~ 6.32)	2.05 (0.70 ~ 6.02)	1.06 (0.55 ~ 2.03)	0.92 (0.41 ~ 2.02)
2nd	1.03 (0.53 ~ 2.00)	0.91 (0.20 ~ 4.06)	1.85 (0.64 ~ 5.37)	0.87 (0.48 ~ 1.59)	0.78 (0.37 ~ 1.64)
3rd	0.65 (0.31 ~ 1.37)	0.8 (0.14 ~ 4.50)	1.06 (0.31 ~ 3.61)	0.62 (0.32 ~ 1.21)	0.65 (0.29 ~ 1.48)
Smoking (current/non & ex-smoker)	0.52 (0.13 ~ 2.10)	2.44 (0.44 ~ 13.63)	0.99 (0.18 ~ 5.49)	0.45 (0.12 ~ 1.71)	1.57 (0.45 ~ 5.41)
Hypertension (/no)	1.26 (0.84 ~ 1.90)	0.69 (0.30 ~ 1.59)	1.17 (0.68 ~ 2.03)	0.86 (0.58 ~ 1.27)	1.02 (0.63 ~ 1.65)
Diabetes (/no)	0.99 (0.51 ~ 1.92)	3.64 (1.36 ~ 9.70)**	1.64 (0.74 ~ 3.63)	0.96 (0.51 ~ 1.81)	1.13 (0.53 ~ 2.41)
Musculoskeletal pain (/no)	7.18 (4.68 ~ 11.02)***	6.13 (2.17 ~ 17.33)***	12.43 (5.79 ~ 26.7)***	6.01 (4.04 ~ 8.93)***	2.62 (1.61 ~ 4.27)***
Stress (/low)	1.53 (0.98 ~ 2.41)	2.31 (1.02 ~ 5.24)*	2.34 (1.31 ~ 4.17)**	1.29 (0.84 ~ 1.99)	4.09 (2.55 ~ 6.56)***
Occupation (/Elementary occupations)	0.65 (0.41 ~ 1.04)	0.78 (0.30 ~ 2.04)	0.46 (0.23 ~ 0.91)*	1.09 (0.71 ~ 1.68)	0.85 (0.50 ~ 1.45)
Working hour/week	0.98 (0.97 ~ 0.99)**	0.98 (0.96 ~ 0.99)*	0.97 (0.96 ~ 0.99)***	0.98 (0.98 ~ 0.99)**	0.99 (0.98 ~ 1.00)
Employment status (/Regular employee)	0.76 (0.48 ~ 1.20)	1.87 (0.66 ~ 5.27)	1.37 (0.71 ~ 2.65)	1.28 (0.83 ~ 1.95)	1.23 (0.72 ~ 2.10)
R^2^_N_	0.473	0.879	0.748	0.382	0.618

Some variables were reclassified because of small group size. Education (0: elementary school, 1: middle and high school, and collage); occupation (0: elementary occupations, 1: office, service and manufacturing work)

*OR* odds ratio, *CI* confidence intervals

*p < 0.05, **p < 0.01, ***p < 0.001

## Discussion

Korea is aging much more rapidly than other countries. The aging includes those in the productive work force. In Korea, the percentage of older workers ≥50 years of age has increased from 22.6 % of all employed persons in 2000 to 27.7 % in 2013. Approximately 77 % of wage-earning wage workers from 2009–2013 was ≥50 years of age [28].

Aging has significant effects on production [29, 30]. The aging of current employees is decreasing their productivity due to the decrease in their physical abilities. Enterprises are trying to restructure to reduce their wage burden on account of the aged, and the vicious cycle of productivity deterioration is likely to continue. Also, with the increase in senile disorders, its cost burden on Korean society is increasing. Regional population is experiencing discrimination due to age, so central and local governments need to formulate policies supporting the welfare of the aged. To achieve this, the health status of current older workers must be determined. Based on this, national government must formulate plans to maintain and extend the working abilities of older workers.

It is important to measure the HRQL of older workers. This information can be used in decisions concerning older worker-related health and medical policies, and can be indicators of the outcomes of related projects. The factors that produced significant differences in the older workers’ EQ-5D index were age, education, household income, cerebro-cardiovascular event, and presence of osteoarthritis, musculoskeletal pain, and stress (Table 3). Past studies of HRQL have identified age, education, income, stress, and number of chronic diseases as factors affecting the EQ-5D index [8–12]. This was confirmed presently. And the associated occupational factors were occupation type, employment status, and working hours (Table 4). In prior studies that analyzed workers’ employment status and HRQL [31], the tendency for the average EQ-5D index to be lower among temporary and daily employees than among regular employees was reported. This was also evident in the present study.

And as seen in Table 2, differences were evident according to gender in the distributions of the occupations and the employment status. More female older workers were engaged in elementary occupations than male older workers, and most of the female older workers were temporary or daily employees rather than regular employees. The data indicate a more unstable working environment for female older workers.

The response frequency at level 1 at each area of EQ-5D was minimum 69.5 % (pain/discomfort with older female workers) and maximum 69.5 % (pain/discomfort with older female workers). Because EQ-5D questionnaire divides the perceived problem levels into three levels at each item, slight problems cannot be reported; this is called as ceiling effect. This has been pointed out as an inherent problem of EQ-5D because the purpose of developing EQ-5D was to measure HRQL easily [32]. Compared with the studies from other countries, it was turned out that 59.2 % (pain/discomfort) at minimum and 95.9 % (self-care) at maximum in America [33] and 78.0 % (pain/discomfort) at minimum and 96.7 % (self-care) at maximum in China [34]. Ceiling effect was seen in this research as well, but it seems to be lower than in that of general population. Ceiling effect of EQ-5D is often pointed out as a flaw of the tool. However, EQ-5D normally shows a high response rate because it is easy to use [34], and ceiling effect tends to decrease in elder population than in general population. With this regard, we believe that it is an appropriate tool to use for a research toward massive group.

The odds ratio (OR) of problems with each of the EQ-5D items was determined with binary logistic regression (Tables 6 and 7). The variables which remained meaningful in both male and female were age and musculoskeletal pain. Age related in all dimensions both male and female, with ORs exceeding 1. It is well known that the probability of problem in EQ-5D increases as the age increases. However, it turned out not to affect as much as expected as ORs were nearly 1.

Musculoskeletal pain showed the highest OR compared with the other factors. Musculoskeletal pain seldom has fatal results, so little attention is paid to early symptoms. However, among the dimensions of EQ-5D, problems were most frequently appealed to ‘pain/discomfort’ dimension (Table 5) and it was indicated that the more older workers feel this kind of pain, the more they appeal their problems in the other EQ-5D dimensions (Tables 6 and 7). Repeated mitigation of the symptoms and consequence muscle deterioration become more deleterious with increasing age. The symptoms can become chronic. This greatly affects productivity due to short-term cessation, absence from work, and significant lessening of the quality of life. Musculoskeletal pain must be continuously managed, especially in older workers.

Presently, stress was closely associated with the ‘anxiety/depression’ dimension among both the male and female older workers. In related prior studies [13], stress has been documented to have more significant effects on HRQL of the female elderly than male elderly. Management of mental health including stress is nonetheless important for older workers.

Smoking among male older workers caused problems in the dimension of ‘mobility’ (Table 6). The average EQ-5D index was lowest among the current smokers, followed by ex-smokers and non-smokers (Table 3). It appears that older workers must be educated on the benefits of not smoking and their smoking habit must be managed.

Compared with the other factors, hypertension did not produce statistically significant changes in its OR, but the OR of diabetes significantly rose in the ‘mobility’ dimension of male older workers (OR 1.94; 95 % CI, 1.05–3.85) and in the ‘self-care’ dimension of female older workers (OR 3.64; 95 % CI 1.36–9.70). Hypertension itself is asymptomatic and blood pressure regulation is relatively easy with medication. However, diabetes should be treated with medication and strict dietary control, and complications can occur easily, so it can be more problematic than hypertension.

Factors closely related to the working conditions included the type of occupations and employment status in male older workers and the type of occupations and the working hours in female older workers. Occupation type related in ‘mobility’ (OR 0.38; 95 % CI 0.22–0.64) and ‘pain/discomfort’ dimension (OR 0.60; 95 % CI 0.38–0.95) of male and in ‘usual activities’ dimension of female (OR 0.46; 95 % CI 0.23–0.91), with ORs < 1. In other dimensions, the average ORs were less than 1, too, nevertheless those were included 1 in confidence interval. This shows that people who fall to the categories of elementary occupations are more vulnerable to their health-related issues. And the ratio of elementary occupations took up the largest part of older workers (Table 2). We will have to come up with countermeasures and methods of care for those vulnerable occupations.

The employment status displayed increased OR in the ‘usual activities’ dimension of the male older workers (OR 2.78; 95 % CI 1.11–6.96). In other dimensions, the average ORs were exceeding 1, yet those were included 1 in confidence interval. Generally, the working conditions of the non-regular workers is less stable than that of the regular workers, and non-regular (temporary and daily) workers are more liable to be assigned dangerous tasks. This may make non-regular workers injured, and it may have made their usual activities bad. Presently, 33 male older workers (4.6 %) responded that they had problems with their ‘usual activities’ dimension. As the sample size was small, the small difference may have been manifest as significant problems. Further study is needed to exclude the selection bias that by chance many of the subjects who had problems with their usual activities were in non-regular employees.

The working hours made the OR <1 in all dimensions except ‘anxiety/depression’ dimension of female older workers. More studies have been addressing the effects of long working hours on health. Long working hours can raise the occurrence of work-related injuries [35] and affect cardiac disease [36–38], sleep disturbance [39], and mental health [40]. In this study, however, unlike prior studies, the longer the working hours were the lower was the probability of occurrence of problems with these dimensions. Those who have problems with these dimensions may have chosen jobs with short working hours because they have realized that long working hours aggravate their symptoms or problems. Or it could be because those who have these types of problems might be not hired in long-time labor market because they were not preferred by the employers. Since the OR is close to 1, it can show an insignificant difference. But, the appropriate number of hours of work can be a means of raising the quality of life of older workers, so it is necessary to examine the effects of the working hours on older workers and to confirm the appropriate working hours.

To sum up the results of this study, to eventually raise the quality of life of older workers through health maintenance and management, it is necessary to manage musculoskeletal diseases including musculoskeletal pain, mental health factors like stress, difficult chronic diseases including diabetes, and living habits like smoking, as well as paying attention to relatively vulnerable workers, such as non-regular employees and female workers.

The present study determined the health status of older workers, established health-related quality of life and associated factors, examined the relationships between the factors known to have effects on HRQL, and determined the extent of the effects. This research is significant because it clarifies HRQL and presents basic data needed to establish related policies.

There are limitations. First, a cross-sectional research method was used. Thus, correlations between variables do not always show causal relationships. Second, the KNHANES results could have been affected by the differences in the subject selection method, questionnaire survey method, and survey period. The EQ-5D survey results could have been affected by the differences in the health levels and the cultural differences in the attitude toward and awareness of health. By supplementing these factors, more in-depth studies are needed concerning diverse and comprehensive disease structures and subjective health status.

## Conclusions

Using the data from the 5th KNHANES, health status, working conditions and HRQL of Korean older workers were examined. Factors that influenced the EQ-5D dimensions of older workers were age, musculoskeletal pain, stress, diabetes, smoking, occupation type, employment status, and working hours.

In this aging era, health management of older workers is absolutely necessary. For efficient health management for older workers, their health must be continuously managed through the development of diverse policies and health promotion programs that reflect sociodemographic characteristics, health-related characteristics, and work-related factors. Studies geared toward increasing the quality of life of older workers should be conducted.
